# L‐Glutamine Plus L‐Glutamic Acid Enhances Antioxidant Status and Ammonia Toxicity Resilience, Upregulates Interleukin *IL‐10* Gene, and Improves Gut Microbiota and Survival in Juvenile Nile Tilapia

**DOI:** 10.1111/jpn.70086

**Published:** 2026-06-28

**Authors:** Cristiana Leonor da Silva Carneiro, Thaís Pereira da Cruz, Larissa Cassemiro Pacheco Monteiro, Larissa Glugoski, Mariana Vieira Feldhaus, Leandro Cavalcante Lipinski, Marcelo Ricardo Vicari, Viviane Nogaroto, Valéria Rossetto Barriviera, Wilson Massamitu Furuya

**Affiliations:** ^1^ Postgraduate Program in Animal Science State University of Maringá Maringá Paraná Brazil; ^2^ Departament of Animal Science State University of Ponta Grossa, Postgraduate Program in Animal Science Ponta Grossa Paraná Brazil; ^3^ Department of Biochemistry and Molecular Biology Postgraduate Program in Applied Biochemistry, Federal University of Viçosa Viçosa Minas Gerais Brazil; ^4^ Postgraduate Program in Animal Science State University of Ponta Grossa Ponta Grossa Paraná Brazil; ^5^ Department of Medicine State University of Ponta Grossa, Postgraduate Program in Animal Science Ponta Grossa Paraná Brazil

**Keywords:** ammonia excretion, anti‐inflammatory cytokine, conditionally essential amino acid, gut health and function, microbiome, oxidative stress status

## Abstract

Glutamine (Gln) and glutamic acid (Glu) are the most abundant free amino acids (AAs) in the fish body. Although classified as non‐essential AAs, their supplementation can be a strategy to optimize the growth performance and health of fish. This study aimed to investigate the effects of dietary Gln and Glu blend on growth performance, biochemical parameters, gut microbiota composition, short‐chain fatty acids (SCFAs) production, digestive enzyme activity, histomorphometry, and liver mRNA levels of *glutamine synthetase* (*GS*), *peroxisome proliferator‐activated receptor alpha* (*PPAR‐α*), anti‐inflammatory *interleukin 10* (*IL‐10*), pro‐inflammatory *interleukin 1β* (*IL‐1β*), and antioxidant status of juvenile Nile tilapia. Fish (*n* = 216; 0.99 ± 0.01 g) were randomly allocated into eight aquariums containing 27 fish each, in a four‐replicate design. Fish were hand‐fed a Glu + Gln unsupplemented basal diet (CON) or a basal diet supplemented with 20 g kg^−1^ Glu + Gln (AMG) six times daily until apparent satiety for 60 days. Relative to fish fed CON diet, fish fed AMG diet exhibited enhanced feed conversion ratio (FCR; −5.6%), energy retention efficiency (+8.20%), and protein retention efficiency (+7.69%), and a trend towards a higher survival rate (+5.9%), suggesting improved nutrient utilization. Although the general structure of the microbiota of fish fed AMG diet remained similar to that of fish fed CON diet, it was observed that Gln + Glu supplementation promoted increased relative abundance of *Enterococcus sp*., a potential probiotic. Notably, fish fed AMG diet showed higher SCFA production than those fed CON diet, enhancing intestinal fold development. Fish fed AMG diet also exhibited higher liver activity of superoxide dismutase (SOD) and glutathione‐S‐transferase (GST), resulting in lower malondialdehyde (MDA) concentration and indicating a healthier intestinal mucosal state. Furthermore, fish fed AMG diet showed higher mRNA expression of *IL‐10* and *GS*, indicating enhanced anti‐inflammatory responses and ammonia metabolism, respectively. In conclusion, 20 g kg^−1^ dietary Gln plus Glu enhanced FCR, nutrient retention, and survival by favorably modulating the microbiota and improving intestinal function, thereby optimizing antioxidant responses and innate immunity in juvenile Nile tilapia. These findings highlight the potential of Gln and Glu blend to improve profitability and sustainability in Nile tilapia aquaculture.

**Abbreviations**
AA: amino acid
ALP: alkaline phosphatase
ALT: alanine aminotransferase
AMG: diet supplemented with a blend of Gln and Glu
AST: aspartate aminotransferase
BSA: bovine serum albumin
CAT: catalase
CEUA/UEPG: Ethics Committee of the State University of Ponta Grossa
CNDB: chloro‐2,4‐dinitrobenzene
CNPq: National Council for Scientific and Technological Development
CON: control diet
DMSO: dimethylsulfoxide
FAS: fatty acid synthase
FCR: feed conversion ratio
Gln: glutamine
Glu: glutamic acid
GS: glutamine synthetase
GSH: glutathione
GST: glutathione‐S‐transferase
HE: hematoxylin‐eosin
HPLC: high‐performance liquid chromatograph
IGM: immunoglobulin
IL‐10: interleukin 10
IL‐1β: interleukin 1β
MDA: malondialdehyde
MS‐222: tricaine methanesulfonate
MTT: 3‐(4,5‐dimethylthiazol‐2‐yl)‐2,5‐diphenyltetrazolium
NMDS: non‐metric multidimensional scaling
OTU: operational taxonomic unit
PPAR‐α: peroxisome proliferator‐activated receptor alpha
RAS: recirculation aquaculture system
SCFA: short‐chain fatty acid
SD: standard deviation
SOD: superoxide dismutase
TBARS: thiobarbituric acid reactive substances
TCA: trichloroacetic acid

## 1 Introduction

Aquaculture faces challenges from environmental stressors and dietary factors promoting chronic inflammation and oxidative stress, which can enable energy and nutrient allocation during critical periods of fish rearing (Dara et al. 2023). Alternatively, amino acids (AAs) such as glutamine (Gln) and glutamic acid (Glu) have gained considerable attention to enhance production efficiency, improve stress management of fish, and promote sustainable aquaculture (Caballero‐Solares et al. 2015; Fuentes‐Quesada et al. 2023; Li et al. 2020; Zhao et al. 2015). These AAs constitute a major portion of free and protein‐bound AAs in the body of fish (Li et al. 2020). Their individual or combined supplementation has been linked to improved growth performance and enhanced gut health and function (Carvalho et al. 2023; Macêdo et al. 2021; Wang et al. 2025). Both AAs play crucial roles in protein synthesis and regulation of key metabolic pathways of fish (Li et al. 2009). Gln acts as a direct precursor of peptides to synthesize proteins, purines, pyrimidines, and nucleic acids, and serves as an indirect precursor of arginine (Hoseini et al. 2020) that improves growth and ammonia detoxification in fish (Chen et al. 2016; Hoseini et al. 2019). Gln can be oxidized to form Glu (Li and Fang. 2009), which is then converted to ammonia and subsequently reconverted to Gln by the enzyme glutamine synthetase (*GS*), helping to reduce ammonia toxicity in fish (Li et al. 2009; Li et al. 2020).

*Peroxisome proliferator‐activated receptor alpha* (*PPAR‐α*) is a nuclear hormone receptor that acts as a regulatory center of glucose, lipid, and AA metabolism (Kersten 2014). For example, previous study reported that upregulated expression of *PPAR‐α* promoted fatty acid oxidation, glycogen accumulation, and muscle protein synthesis in mice (Makowski et al. 2009). Additionally, *PPAR‐α* has been proven to suppress *GS* expression in rat liver by inhibiting Gln catabolism, preventing ammonia production, and redirecting ammonia for protein synthesis (Newsholme et al. 2003; Velázquez‐Villegas et al. 2016). Thus, evaluating the *GS* and *PPAR‐α* gene expression in Nile tilapia (*Oreochromis niloticus*) fed Gln and Glu‐supplemented diets could provide valuable insights into ammonia detoxication and energy metabolism. A recent study conducted in our laboratory found that Gln and Glu blend used herein up‐regulates gene expression of *GS* and *PPAR‐α* in Nile tilapia fingerlings (Carneiro et al. 2025). However, the mechanisms underlying this regulation should be explored in other Nile tilapia growing phases, considering that the metabolic and ammonia detoxification capacity of fish may vary ontogenetically (Gao et al. 2020).

Early studies attributed the improvements in growth and intestinal morphology in Nile tilapia to Gln and/or Glu dietary supplementation (Carvalho et al. 2023; Graciano et al. 2014; Macêdo et al. 2021). These benefits stem from the high metabolic demand of enterocytes for these AAs as a primary energy source (McCauley et al. 1998). Furthermore, previous studies have shown that Gln positively modulates the gut microbiota in turbot, *Scophthalmus maximus* (Gu et al. 2017; Liu et al. 2018), potentially playing a role in maintaining intestinal health. Gut bacteria can ferment complex carbohydrates, responsible for the production of short‐chain fatty acids (SCFAs), which play a critical role in favoring gut morphology and function (Cruz et al. 2024). For example, previous studies confirmed that Gln or Glu increased the relative abundance of fermentative bacteria responsible for SCFA production in the gut of pigs (Hu et al. 2019; Yan et al. 2020), which in turn reduces the expression of inflammatory cytokines in fish, as demonstrated in common carp, *Cyprinus carpio* (Petit et al. 2022). However, further study is required to elucidate the mechanisms by which Gln and Glu modulate gut microbiota and SCFA production in juvenile Nile tilapia.

Recent studies have also demonstrated that Gln and/or Glu play a vital role in activating the immune response (Carvalho et al. 2023, 2018; Li et al. 2023) and improving the antioxidant response in Nile tilapia (Carvalho et al. 2023; Wang et al. 2025). Lymphoid organs demand large amounts of Gln to supply energy for lymphocyte proliferation and support macrophage phagocytosis (Calder and Yaqoob 1999), resulting in the production of cytokines, particularly pro‐inflammatory *interleukin‐1β* (*IL‐1β*) and anti‐inflammatory *interleukin‐10* (*IL‐10*), as previously demonstrated in humans (Coëffier et al. 2001) and also in fish species such as yellow catfish, *Pelteobagrus fulvidraco* (Jiao et al. 2022) and turbot (Gu et al. 2017). On the other hand, Glu acts as a precursor of glutathione (GSH), a compelling intracellular antioxidant in fish (Li et al. 2020). Consequently, Glu sustains GSH homeostasis and induces the activity of other antioxidant enzymes, including superoxide dismutase (SOD) and catalase (CAT), thereby improving fish antioxidant status (Coutinho et al. 2016), as observed in Nile tilapia juveniles (Carvalho et al. 2023).

In a recent study conducted in our laboratory, it was also found that the Gln and Glu blend increased *IL‐10* gene expression and modulated oxidative responses in Nile tilapia fingerlings (Carneiro et al. 2025). However, the effects of mixing Gln and Glu on the expression of interleukins *IL‐10* and *IL‐1β*, as well as on the oxidative status of fish, remain poorly documented in later growth phases, given the marked differences in growth rates across the developmental stages. Therefore, this study extends our previous work on fingerling Nile tilapia (Carneiro et al. 2025) by investigating the effects of dietary Gln and Glu supplementation on growth performance, plasma biochemical parameters, intestinal histomorphometry, digestive enzyme activities, SCFA production, and microbiota composition in juvenile Nile tilapia. Furthermore, the study evaluated the effects of the Gln–Glu blend on the expression of genes associated with AA metabolism, including *GS* and *PPAR‐α*, inflammatory cytokines *IL‐1β* and *IL‐10*, and antioxidant status of fish.

## 2 Materials and Methods

### 2.1 Ethics Statement

This research was approved by the Ethics Committee of the State University of Ponta Grossa (CEUA/UEPG) under protocol (23.000022613‐5). This study is part of a project to evaluate the effects of Gln and Glu blend dietary supplementation across different growth phases of Nile tilapia: fingerling (Carneiro et al. 2025), juvenile, and adult. For euthanasia, the fish were immersed in a water solution (1 L) containing 400 mg L^−1^ of tricaine methanesulfonate (MS‐222; Sigma‐Aldrich, São Paulo, Brazil) until total loss of consciousness. Afterward, a qualified person applied the physical penetrating captive bolt method (AVMA 2020). The dosage of MS‐222 for euthanasia was established as fourfold the dosage used for anesthesia (AVMA 2020).

### 2.2 Diet Formulation and Preparation

A basal diet, to be used as the control diet (CON), was formulated to meet the dietary requirements of juvenile Nile tilapia in terms of energy and protein (Abdel‐Tawwab et al. 2010), energy/protein ratio (Peres et al. 2022), AAs (Furuya et al. 2023) and phosphorus levels (Yao et al. 2014), as described in Tables 1 and 2.

**Table 1:** Ingredients and analyzed composition of experimental diets.

Ingredient (g kg^−1^; as fed basis)	Diet^a^	
	CON	AMG
Soybean meal^b^	191	191
Fish meal^b^	151	151
Rice flour^b^	123	123
Spray‐dried plasma^b^	40	40
Poultry by‐products^b^	390	390
Soybean oil^b^	20	20
Corn gluten meal 60^b^	60	60
Mineral and vitamin mix^c^	5	5
l‐alanine^d^	12.2	0
AminoGut^e^	0	20
Inert (Silica)^f^	7.8	0
Analyzed composition (g kg^−1^; Dry matter)		
Dry matter	934.6	924.8
Gross energy (MJ kg^−1^)	22.3	22.9
Crude protein	472.4	477.3
Crude lipids	80.8	80.4
Crude fiber	21.1	22.6
Ash	81.2	82.4
Calcium	18.6	17.4
Total phosphorus	11.1	11.6

^a^ Diet unsupplemented (CON) or supplemented with blend of l‐glutamine and l‐glutamic acid at 20 g kg^−1^;

^b^ Supplied by Alisul Alimentos S.A. Supra, Maringá,Brazil;

^c^ Customized premix (Composition per kilogram of feed (IU or mg kg^−1^ of diet): Vitamin A, 15,000 IU; vitamin D_3_, 5000 IU; vitamin E (DL‐α‐tocopheryl acetate), 100 mg; vitamin K_3_, 12 mg; vitamin B_1_, 24 mg; vitamin B_2_, 24 mg; vitamina B_3_, 160 mg; vitamina B_5_, 100 mg; vitamin B_6_, 34 mg; vitamin B_8_, 1.8 g; vitamin B_9_, 8.5 mg; vitamin B_12_, 24 mg; ascorbic acid, 750 mg; iron, 50 mg; copper, 6 mg; manganese, 20 mg; zinc, 100 mg; iodine, 0.4 mg; selenium, 0.3 mg; butylated hydroxytoluene, 0.2 g; calcium propionate, 1 g;

^d^ Ajinomoto Animal Nutrition Division, São Paulo, Brazil);

^e^ Blend of l‐glutamine (minimum 10%) and l‐glutamic acid (minimum, 10%) (Ajinomoto Animal Nutrition Division, São Paulo, Brazil);

^f^ Sigma Aldrich, São Paulo, Brazil.

**Table 2:** Analyzed amino acid composition of the experimental diets (g kg^−1^ dry‐matter basis).

Item	Diet^a^	
	CON	AMG
Indispensable amino acids		
Arginine	23.4	22.2
Histidine	11.7	11.6
Isoleucine	13.2	13.4
Leucine	43.9	43.1
Lysine	21.1	21.2
Methionine	9.4	9.1
Phenylalanine	21.4	20.9
Threonine	16.3	15.8
Tryptophan	4.1	3.6
Valine	22.6	22.6
Dispensable amino acids		
Alanine	41.4	29.8
Aspartic acid	33.4	33.0
Cystine	6.2	6.0
Glutamine + Glutamic acid	58.0	74.6
Glycine	29.6	29.3
Serine	21.4	21.1
Tyrosine	13.5	13.9

^a^ Diets were either unsupplemented (CON) or supplemented (AGM) with a blend of l‐glutamine and l‐glutamic acid at 20 g kg^−1^.

The AMG diet was established by supplementing the CON diet with 20 g kg^−1^ of Gln (l‐glutamine) and Glu (l‐glutamic acid), supplied as the commercial blend AminoGut (Ajinomoto do Brasil Indústria e Comércio de Alimentos Ltda, Animal Nutrition Business Unit, São Paulo, Brazil). Gln + Glu was added at the expense of l‐alanine in the CON diet to obtain isonitrogenous diets. Considering the analyzed nitrogen levels of l‐alanine converted to 610.7 g kg^−1^ of protein, the commercial product was included at 12.2 g kg^−1^, ensuring nitrogen equivalence. AminoGut contains guaranteed minimum levels of 10% l‐glutamine and 10% l‐glutamic acid and an analyzed nitrogen level equivalent to 993.8 g kg^−1^ of protein. Silica was subsequently added to complete 1000 g of diet.

All ingredients were finely ground (0.5 mm) in a centrifugal mill (Vieira MC680B, Tatuí, Brazil) and passed through a sieve. The extrusion of the diets was performed by Alisul Alimentos S. A. ‐ Supra (Maringá, Brazil) in a single‐screw extruder (Exteec EX30, Ribeirão Preto, Brazil) coupled to a drying drum with a temperature set at 85°C for 24 min (Model E‐62, Ferraz Máquinas e Engenharia LTDA, Ribeirão Preto, Brazil). The pellets of 0.8 mm were stable, so 98% of the pellets floated. The top‐coating addition of l‐glutamine and l‐glutamic acid mixture was performed using a commercial vehicle (Vansil Indústria Comércio e Representações Ltda, Descalvado, SP, Brazil). l‐glutamine and l‐glutamic acid mixture was dissolved in 28 mL of commercial vehicle and top‐sprayed onto each kilogram of extruded diet. The same procedure was performed for the CON diet, with l‐alanine used in place of the vehicle.

### 2.3 Fish and Experimental Design

The experiment was conducted at the Aquaculture Laboratory of the State University of Ponta Grossa (Ponta Grossa, Brazil). A total of 50‐day‐old, all‐male masculinized (*n* = 1000; 0.4 ± 0.08 g; mean ± standard deviation; SD), were purchased from AquaGenetics do Brasil Ltda (Porto Ferreira, Brazil) and acclimatized for 3 weeks in 2‐150‐L circular fiberglass aquaria kept in recirculating aquaculture system (RAS) with constant temperature (28°C ± 1°C), dissolved oxygen supply (6.1 ± 1 mg L^−1^) and mechanical and biological filters. Fish were hand‐fed to apparent visual satiety with the basal diet used in the subsequent experimental trial six times a day and 7 days a week. At the end of the acclimatization period, 216 fish (0.99 ± 0.01 g; mean ± SD) were randomly allocated into eight 100 L rectangular plastic aquariums (40 cm long × 40 cm wide × 62.5 cm high) arranged in another RAS, at a density of 27 fish per aquarium. This RAS, with a water flow rate of 1.4 L min^−1^ per aquarium, contained a solid waste decanter filter with plastic media, a 55‐W UV light filter, a 2000‐W heater coupled with digital thermostat, a 1500 L h^−1^ submerged water pump, and a 0.38‐HP centrifugal blower (Sulpesca, Toledo, Brazil) coupled with 1.5‐mm silicone airline tubing with porous stones in each aquarium to provide aeration. A microbubble diffused‐air system was inserted into the mechanical filter to increase the dissolved oxygen concentration in the RAS. Water quality parameters were daily monitored. Throughout the experiment, the water temperature was kept at 28°C ± 1°C and dissolved oxygen at 6.0 ± 0.3 mg L^−1^ (YSI Multi‐Parameter Water Quality Meter, YSI Incorporated, Ohio, USA). The pH (TEC‐2, Piracicaba, Brazil) and alkalinity (Hanna, HI775, Tamboré, Barueri, Brazil) were kept at 6.8 and 60 mg L^−1^, respectively, by adding calcium carbonate and/or phosphoric acid in the RAS as needed. Non‐ionized ammonia and nitrite (Alfakit, Florianópolis, Brazil) were kept below 0.05 and 0.5 mg L^−1^, respectively, by exchanging the water in the RAS. Fish were hand‐fed until apparent visual satiety, six times daily at 8:20, 11:20, 13:20, 14:20, 16:20, and 18:20 h, 7 days a week, for 60 days. Feeding was adjusted to ensure complete feed intake within 120 s (Pedrazzani et al. 2020). Any uneaten feed remaining after 160 s of the feed period was collected with a propylene sieve, dried in a ventilated oven at 55°C for 24 h, and weighed to ascertain the feed intake.

### 2.4 Sample Collection

At the beginning of the feeding trial, approximately 40 fish were randomly sampled to analyze the body composition. On day 60 of the experimental period, fish from each aquarium were fasted for 24 h, weighed in groups, and counted. Fish were then returned to their respective aquariums and fed staggered (Cruz et al. 2024). Then, 4 h after feeding, three fish per aquarium were randomly collected and euthanized. Fish were dissected under aseptic conditions to obtain digesta samples for analysis of microbiota composition (~750 mg) and SCFA (~750 mg). Digesta samples were obtained from the midgut portion of the intestine by gently compressing and collecting with a micropipette. Then, digesta samples from the three fish were pooled in a 2 mL cryogenic tube and immediately frozen in liquid nitrogen until final storage at −80°C (Brito et al. 2022). For digestive enzyme activity, a 3 cm section of midgut from the same three fish was collected from each fish and pooled in a 2 mL cryogenic tube, immediately frozen in liquid nitrogen, and subsequently stored at −80°C until analysis (Cruz et al. 2024). Finally, liver samples (~0.5 g) from each three fish were collected for gene expression and evaluation of antioxidant status and stored in a 2 mL cryogenic tube, frozen in liquid nitrogen, and subsequently stored at −80°C until analysis.

After the first day of sample collection, the remaining fish were fasted for 24 h, then counted and weighed as a group. Subsequently, six fish from each aquarium were randomly collected and euthanazied for analysis of total body composition and stored at −20°C until analysis. Three other fish from each aquarium were randomly collected and anesthetized for blood sample collection using MS‐222 solution at 100 mg L^−1^ (Osei et al. 2022). Blood (~1 mL) from each of the three fish was gently withdrawn from the caudal vein using 1 mL heparinized syringes, pooled, and centrifuged at 1200*g* for 10 min using a microcentrifuge (Kasvi–SKU K14‐1215, São José dos Pinhais, Brazil). From the same three fish, approximately 1 cm of midgut segment was collected for histomorphometric analysis. For this purpose, the intestinal tissue was fixed in 10% buffered formalin for 24 h. Finally, the weight of the liver and visceral fat of these fish were recorded for the determination of somatic indices.

### 2.5 Chemical Analysis

Whole‐body and experimental diets were analyzed following standard methods of the Association of Official Analytical Chemists (AOAC 2006). First, moisture content was obtained by oven‐drying the samples at 105°C for 72 h. Crude protein (*N* × 6.25) content was determined following the macro Kjeldahl method after acid hydrolysis (Tecnal, MA‐036, Piracicaba, Brazil). Crude lipid analysis followed the chloroform: methanol method (Folch et al. 1957). Crude fiber was analyzed following the loss on ignition of dried lipid‐free residues, using acid digestion with 1.25% H_2_SO_4_ and basic digestion with 1.25% NaOH, respectively. Ash was measured by overnight combustion in a muffle furnace at 550°C (Tecnal, 2000B, Belo Horizonte, Brazil). Calcium and phosphorus contents were determined by using the inductively coupled plasma optical emissions spectrometry (ICP‐OES) method using an internally validated analysis method (PerkinElmer 8000, Waltham, USA), as previously established (Schamber et al. 2014). Finally, the gross energy analysis was determined using an adiabatic calorimeter bomb (Parr 6400; Parr Instruments Co., Moline, USA), employing benzoic acid as a calibration standard. All AA contents were determined by using a High‐Performance Liquid Chromatograph (HPLC) (Hitachi, Tokyo, Japan) after acid hydrolysis (Rayner 1985). Separately, dietary tryptophan analysis was determined using the alkaline hydroxylation method with lithium hydroxide.

### 2.6 Calculations

Growth performance parameters were calculated as follows:

- Final body weight (g) = total fish weight (g) ÷ number of fish;
- Body weight gain (g) = final body weight (g) – initial body weight (g);
- Fish biomass (g) = final body weight of fish × number of fish per aquaria (g);
- Absolute feed intake (g) = total feed consumed (g);
- Feed conversion ratio (FCR) = feed consumed (g) ÷ body weight gain (g);
- Energy retention efficiency (%) = {[(final body weight (g) × final body gross energy (kJ)) – (initial body weight (g) × initial body gross energy (kJ))] ÷ gross energy consumed (kJ)} × 100;
- Protein retention efficiency (%) = {[(final body weight (g) × final body crude protein (g)) – (initial body weight (g) × initial body crude protein (g))] ÷ crude protein consumed (g)} × 100;
- Hepatosomatic index (%) = (liver weight (g) ÷ final body weight (g)) × 100;
- Visceral fat ratio (%) = (visceral fat weight (g) ÷ final body weight (g)) × 100;
- Survival (%) = (final number of fish ÷ initial number of fish) × 100.

### 2.7 Plasmatic Parameters

Plasma levels of glucose (Cat. 90.017.00), total protein (Cat. 90.019.00), triacylglycerol (Cat. 90.022.00), cholesterol (Cat. 90.021.00), alanine aminotransferase (ALT; Cat. 90.013.00), aspartate aminotransferase (AST; Cat. 90.015.00) and alkaline phosphatase (ALP; Cat. 90.015.00) were determined by spectrometry in a semiautomatic biochemical analyzer (BIO‐2000 IL, Barueri, Brazil) using commercial kits (Bioclin–Quibasa, Belo Horizonte, Brazil).

### 2.8 Gut Microbiota

DNA from digesta samples was extracted using the GenElute Soil DNA Isolation Kit (Sigma‐Aldrich, St. Louis, USA) following the manufacturer's protocol. Amplification of the V4 hypervariable region of the 16S ribosomal rRNA gene was performed using the universal primers 515 F and 806 R. PCR conditions include an initial denaturation (94°C for 3 min), followed by denaturation (18 cycles of 94°C for 45 s), annealing (50°C for 30 s), and extension (68°C for 60 s). A final elongation step was performed at 72°C for 10 min to ensure complete amplification. The resulting amplicons were sequenced on an Illumina “MiSeq” platform (Degnan and Ochman 2012). The reads were processed and analyzed using the QIIME2 (Quantitative Insights Into Microbial Ecology) platform. The process began with the removal of low‐quality sequences, followed by filtering and removal of chimeras, and subsequent taxonomic classification of the remaining sequences (Caporaso et al. 2011). Taxonomic assignment (> 97% similarity) was performed by aligning sequences against reference genomes from the SILVA database, version 138.2 (Yilmaz et al. 2014). To ensure robust comparisons, bacterial community classification was conducted using normalized data, preventing taxonomic biases associated with variations in sequencing depth. On average, 15,742 high‐quality reading counts per sample were used for the analysis.

### 2.9 Activity of Digestive Enzymes

Midgut samples with digesta were homogenized by cell rupture in a liquid nitrogen bath with subsequent addition of HCl buffer solution, pH 3. The extracts obtained were centrifuged at 14,000*g* for 30 min at 4°C. The supernatants were collected to determine the total protein concentration and the activities of amylase, total proteases, and lipase. The protein concentration in the samples was determined using bovine serum albumin (BSA) as a standard, following the method proposed by Bradford (1976).

Total proteolytic activity was assessed following a casein hydrolysis method (Kunitz 1946) with modifications (Walter 1984). The enzymatic reaction used a mixture of 0.25 mL of 1% casein, 0.25 mL of 0.1 M Tris‐HCl (pH 7), and 0.1 mL of the enzymatic extract obtained from the samples. The reaction was incubated at 35°C for 30 min, after which 0.6 mL of 8% trichloroacetic acid (TCA) was added to terminate the reaction. Samples were incubated at 2°C for 1 h to allow protein precipitation, then centrifuged at 1800*g* for 10 min. The absorbance of the resulting solutions was measured at 340 nm.

Enzymatic activity was quantified using tyrosine as a standard, with 1 unit defined as the amount of enzyme required to catalyze the production of 1 µg min^–1^ of tyrosine. Amylase and lipase activities were assessed using the commercial kits K046‐1.1 and K093‐12, respectively (Bioclin Quibasa, Belo Horizonte, Brazil). The analyses were performed following the manufacturer's protocols, according to the methodologies described by Cherry and Crandall (1932) for amylase and Caraway (1959) for lipase.

### 2.10 Digesta pH and SCFAs Concentration

The pH of the gut digesta was measured using a portable digital pH meter (Kasvi–ATC‐K39‐0014PA, São José dos Pinhais, Brazil) inserted directly into the digesta. The gut acetic, propionic, and butyric acid concentrations were determined using a gas chromatograph (Shimadzu GC‐2010 Plus chromatograph, Tokyo, Japan) equipped with an automatic injector (AOC‐20i) and a flame ionization detector. Before analysis, samples were acidified with 1 M o‐phosphoric acid (P.A.) (Ref. 100573, Merck) and a mixture of free volatile acids (Ref. 46975, Supelco). Separation was performed using a Stabilwax‐DA capillary column (30 m, 0.25 mm ID, 0.25 μm df, Restek). Afterward, a 1 μL aliquot was injected (40:1) with helium as the carrier gas at a 42 cm s^–1^ flow rate. The chromatographic run lasted 11.5 min, with a temperature of 250°C and a detector temperature of 300°C. The column temperature program included an initial hold at 40°C, followed by a gradient increase to 120°C at 40°C min^−1^, then to 180°C at 10°C min^−1^, and finally to 240°C at 120°C min^−1^, where it was maintained for 3 min. Calibrated dilutions of the WSFA‐2 standard (Ref. 47056, Supelco) and glacial acetic acid (Ref. 33209, Sigma‐Aldrich) were analyzed under these conditions. Peak identification and integration were performed using GCsolution software version 2.42.00 (Shimadzu, Kyoto, Japan).

### 2.11 Gut Histomorphometry

Intestine sections were mounted in paraffin blocks (Prophet 1992) and sectioned transversely to obtain 5 µm thick sections. The sections were stained with hematoxylin‐eosin (HE) (Dimitroglou et al. 2010) and mounted on histological slides. Three histological sections per fish were captured using an optical microscope with a Pro‐Seires camera (Media Cybertechniques, Olympus, Tokyo, Japan).

The heights and widths of the intestinal folds were evaluated by measuring 100 undamaged folds of each fish, totaling 1600 measurements per treatment. The measurements were made using the Image‐Pro Plus program (version 5.2‐CyberMedia). The absorption area of the fold was calculated by multiplying the fold height by the fold width according to the method proposed by Adeshina et al. (2021).

### 2.12 Gene Expression

Total RNA was extracted from the liver tissue using *TRIzol* reagent (Invitrogen, Life Technologies, Carlsbad, USA) following the manufacturer's instructions. cDNA synthesis was performed using 2 µg of total RNA, reverse transcribed with the GoScript Reverse Transcription System (Promega), according to the manufacturer's protocol. Primer pairs for *GS*, *PPAR‐α*, *IL‐1β*, *IL‐10*, and 18S ribosomal DNA (rDNA) were designed based on the cDNA nucleotide sequence from *O. niloticus*, available in GenBank (http://www.ncbi.nlm.nih.gov/pubmed/nucleotide) (Table 3) (Carneiro et al. 2025). Primer efficiency was evaluated using standard curve analysis. For this, a serial dilution of cDNA (starting at 20 ng) was prepared at the following concentrations: ×1, ×0.1, ×0.01, ×0.001, and ×0.0001. Acceptable efficiency values ranged between 90% and 110%, corresponding to a slope between −3.58 and −3.10. The qPCR reaction mixture consisted of ×2 GoTaq qPCR Master Mix (Promega), 0.3 µM of forward and reverse primers, 20 ng µL^−1^ of cDNA, and nuclease‐free water to a final volume of 20 µL. The qRT‐PCR protocol included an initial denaturation at 95°C for 10 min, followed by 40 cycles consisting of denaturation at 95°C for 15 s, annealing at 60°C for 30 s, and extension at 72°C for 30 s. The reaction was concluded with a dissociation curve analysis to verify specificity. Quantitative Real Time‐Polymerase Chain Reaction (qRT‐PCR) was conducted in AriaMx Real‐Time PCR System (Agilent Technologies, Santa Clara, CA, USA). Once primer efficiency was confirmed, qRT‐PCR was performed in duplicate, using ×2 GoTaq qPCR Master Mix (Promega), 0.3 µM of forward and reverse primers, 20 ng of cDNA, and nuclease‐free water to reach a final volume of 20 µL. The thermal cycling conditions were identical to those used for primer efficiency testing. Gene expression levels were normalized to the housekeeping gene 18S ribosomal DNA (18SrDNA).

**Table 3:** qPCR primers of metabolic and immune genes of juvenile Nile tilapia.

Gene	Nucleotide sequence (5′–3′)	Eff%	Slope
*GS*	F: CAGCAACACATTGGACCTGC	98.36%	−3.36
	R: CTGGCGGATGTAGCCATCTT		
*PPAR‐α*	F: ACGGTGTTTACGAAGCCCTC	100.48%	−3.31
	R: TGATAAAGCCTCCACCACGG		
*IL‐10*	F: ACTTTGAGACGTACCTGGCTT	90.10%	−3.58
	R: TGATTTGGGTCAGCAGGTCTT		
*IL‐1β*	F: GGACACGGAAAGGATTGACAG	104.39%	−3.22
	R: GTTCGTTATCGGAATTAACCAGAC		

Abbreviations: 18SrDNA, 18S ribosomal DNA; Eff%, amplification efficiency; *GS*, *glutamine synthase*; *IL‐10*, interleukin 10; IL‐1β, *interleukin 1 beta*; *PPAR‐α*, *peroxisome proliferator‐activated receptor alpha*. Carneiro et al. (2025).

### 2.13 Antioxidant Activity

The liver samples were homogenized with phosphate buffer (pH 7.4) containing EDTA. The mixture was centrifuged at 17,800*g* at 4°C for 10 min, and the supernatant was collected to evaluate the activity of the antioxidant enzymes SOD, CAT, glutathione‐S‐transferase (GST), as well as to determine the concentration of the product of lipid peroxidation, malondialdehyde (MDA). The SOD activity was determined according to the method described by Dieterich et al. (2000) with minor modifications. For this purpose, 3‐(4,5‐dimethylthiazol‐2‐yl)‐2,5‐diphenyltetrazolium (MTT) and pyrogallic acid were added to the samples. After incubation at 40°C for 15 min, dimethyl sulfoxide (DMSO) was added to the mixture, which was then pipetted into microplates, and a reading was taken at 570 nm. The results were expressed as SOD units per milligram of protein. The CAT activity was determined using the method described by Aebi (1984). For this purpose, hydrogen peroxide was added to the sample, and the absorbance values were measured in a spectrophotometer 60 s later at 240 nm. The results were expressed in CAT units per milligram of protein. The GST activity was determined by the method described by Habig et al. (1974), in which 1‐chloro‐2,4‐dinitrobenzene (CNDB) and GSH were added to the sample, and the absorbance at 340 nm was recorded 30 and 90 s later. The GST activity results were expressed in micromoles per minute per gram of tissue units. The MDA quantification was performed using the method described by Buege and Aust (1978). The sample reacted with thiobarbituric acid reactive substances (TBARS) composed of 15% TCA, 0.375% thiobarbituric acid, and 6% hydrochloric acid, and was heated in a water bath (90°C) for 40 min. Subsequently, the mixture was cooled and centrifuged at 14,000*g* for 5 min. Finally, the supernatant was collected and analyzed using a microplate reader at 535 nm. The result was quantified in micromoles per milligram of protein.

### 2.14 Statistical Analysis

All data are presented as mean ± standard error (SE). Data normality was evaluated using the Kolmogorov–Smirnov test, while homogeneity of variance was assessed with Levene's test. Differences between the two treatments were assessed using Student's *t*‐test at a significance level of *p* < 0.05 using the statistical package of Minitab Inc., State College, PA, USA. Intestinal microbiota analysis was achieved using the Microbiome Analyst web server and the R package. Data were filtered and normalized for graphical representation. The normality of alpha diversity metrics was evaluated using the Kolmogorov–Smirnov test, which confirmed that the data did not follow a Gaussian distribution. Thus, differences in the alpha diversity index were analyzed using the Mann−Whitney test. Beta diversity was analyzed using Bray‐Curtis dissimilarities (PERMANOVA) and visualized by non‐metric multidimensional scaling (NMDS). The Venn diagram was constructed considering operational taxonomic units (OTUs) found in a treatment, defined as having a value ≥ 2 in at least three replicates. Differential relative abundance analysis at the genus level was performed using DESeq2 with Benjamini‐Hochberg FDR correction (Calgaro et al. 2020). Relative gene expression was quantified using the comparative CT method ($2^{-\Delta \Delta C_{T}}$) after validation (Livak and Schmittgen 2001). Statistical significance was set at *p* < 0.05, whereas *p* values between 0.05 and 1.00 indicated trends.

## 3 Results

### 3.1 Growth Performance

The effects of dietary treatments on the growth performance of juvenile Nile tilapia are shown in Table 4. Fish fed the AMG diet displayed a tendency for higher fish biomass (*p* = 0.052), enhanced FCR (*p* = 0.015), higher protein retention efficiency (*p* = 0.022) and energy retention efficiency (*p* = 0.022), and a tendency for higher survival ratio (*p* = 0.083) than those fed the CON diet. However, dietary treatments did not affect (*p* > 0.05) final body weight, body weight gain, feed intake, hepatosomatic index, and visceral fat ratio.

**Table 4:** Growth performance of juvenile Nile tilapia fed the experimental diets for 60 days^c^.

Parameter	Diet^d^		*p* value
	CON	AMG	
Initial body weight (g)	0.99 ± 0.01	0.99 ± 0.01	0.731
Final body weight (g)	93.78 ± 2.04	97.37 ± 0.82	0.151
Body weight gain (%)	9361.81 ± 151.52	9674.80 ± 106.36	0.142
Fish biomass (g)	3961.44 ± 80.31	4360.11 ± 144.03	0.052
Absolute feed intake (g)	90.58 ± 1.52	88.87 ± 0.87	0.369
Feed conversion ratio	0.98 ± 0.01^a^	0.92 ± 0.01^b^	0.015
Energy retention efficiency (%)	25.24 ± 1.86^b^	27.31 ± 0.49^a^	0.022
Protein retention efficiency (%)	32.11 ± 0.47^b^	34.58 ± 0.64^a^	0.023
Hepatosomatic index (%)	2.75 ± 0.15	2.71 ± 0.04	0.835
Visceral fat ratio (%)	2.21 ± 0.12	2.11 ± 0.13	0.582
Survival (%)	82.85 ± 0.93	87.75 ± 2.16	0.083

^a‐b^ Different letters in the same row indicate significant differences by *t*‐test (*p* < 0.05);

^c^ Values are presented as means and standard errors of the means of four replicate aquaria (*n* = 27 fish per aquarium);

^d^ Diets were either unsupplemented (CON) or supplemented (AMG) with a blend of l‐glutamine and l‐glutamic acid at 20 g kg^−1^.

### 3.2 Whole‐Body Composition

Table 5 shows the effects of dietary treatments on whole‐body composition. Dietary treatments did not affect whole‐body moisture, gross energy, crude protein, crude lipids, and ash contents (*p* > 0.05).

**Table 5:** Whole‐body proximate composition (g 100 g^−1^ wet weight) of juvenile Nile tilapia fed the experimental diets for 60 days^a^.

Parameter	Diet^b^		*p* value
	CON	AMG	
Moisture	73.69 ± 0.53	74.02 ± 0.51	0.677
Gross energy (kJ)	35.98 ± 1.04	33.90 ± 0.55	0.456
Crude protein	15.22 ± 0.10	15.32 ± 0.18	0.662
Crude lipids	6.97 ± 0.17	6.80 ± 0.13	0.475
Ash	3.01 ± 0.40	3.55 ± 0.13	0.250

^a^ Values are presented as means and standard error of the means of four replicate aquaria (*n* = 6 fish per aquarium).

^b^ Diet were either unsupplemented (CON) or supplemented (AMG) with a blend of l‐glutamine and l‐glutamic acid at 20 g kg^−1^.

### 3.3 Plasma Biochemistry

The effects of dietary treatments on plasma biochemical response are shown in Table 6. The activity of ALP was higher (*p* = 0.019) in fish fed the AMG diet than in those fed the CON diet. Nevertheless, dietary treatments did not affect total protein, glucose, triacylglycerol, and cholesterol concentrations, and the activity of ALT and AST enzymes (*p* > 0.05).

**Table 6:** Plasma biochemical responses of juvenile Nile tilapia fed the experimental diets for 60 days^c^.

Parameter	Diet^d^		*p* value
	CON	AMG	
Total protein (g dL^−1^)	2.5 ± 0.0	2.5 ± 0.1	0.810
Glucose (mg dL^−1^)	79.5 ± 1.0	81.0 ± 0.2	0.217
Triacylglycerol (mg dL^−1^)	388.0 ± 82.8	268.5 ± 88.4	0.362
Cholesterol (mg dL^−1^)	90.7 ± 5.5	84.5 ± 4.3	0.399
Alanine aminotransferase (U L^−1^)	532.3 ± 103.3	706.8 ± 95.3	0.263
Aspartate aminotransferase (U L^−1^)	593.3 ± 138.5	1094.8 ± 244.6	0.125
Alkaline phosphatase (U L^−1^)	633.6 ± 3^b^	901.3 ± 78.6^a^	0.019

^a‐b^ Different letters in the same row indicate significant differences by *t*‐test (*p* < 0.05);

^c^ Values are presented as means and standard error of the means of four replicate aquaria (*n* = 3 fish per aquarium);

^d^ Diets were either unsupplemented (CON) or supplemented (AMG) with a blend of L‐glutamine‐glutamine and l‐glutamic acid at 20 g kg^−1^.

### 3.4 Gut Microbiota

**Figure 1:**
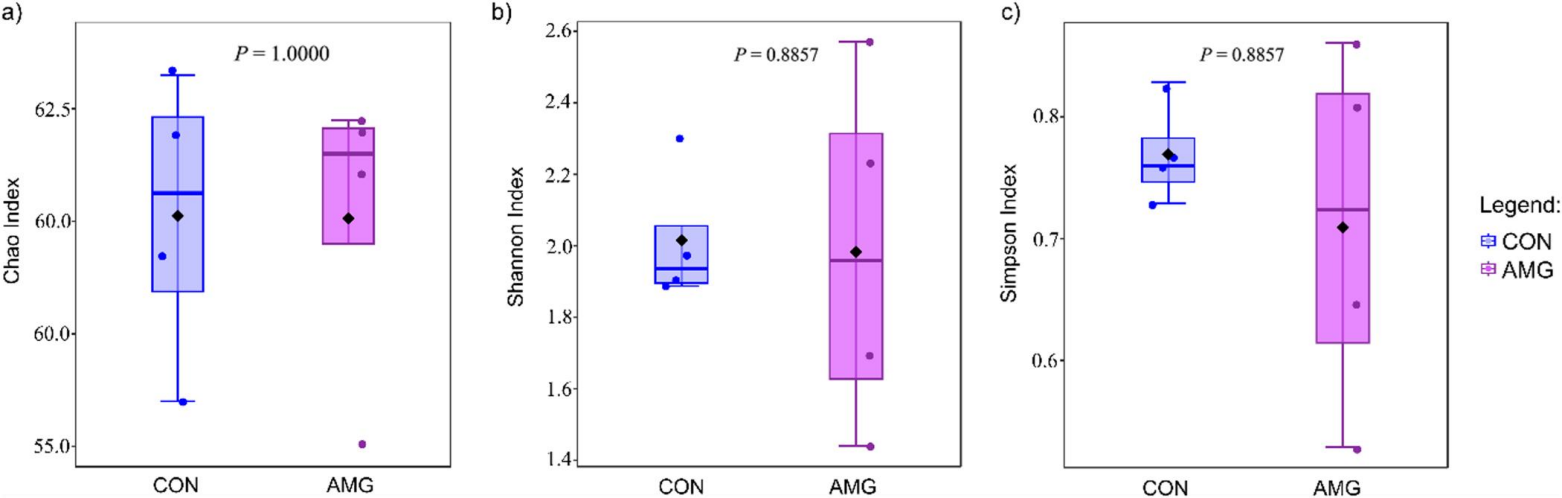
Alpha diversity assessed by Chao (a), Shannon (b), and Simpson (c) indices in juvenile Nile tilapia fed diets either without supplementation (CON) or supplemented with a blend of l‐glutamine and l‐glutamic acid at 20 g kg^−1^ as AminoGut (AMG), over a 60‐day feeding period. Data for each treatment represent the mean of four replicate aquaria. [Color figure can be viewed at wileyonlinelibrary.com]

The sequencing process produced 372,450 reads, averaging 46,556 reads per sample, with a minimum of 15,742 reads. As illustrated, the rarefaction curves reached the saturation plateau, and Good's coverage for all samples was 0.998, confirming sequencing depth (Supporting Information S1: Figure 1).

The impact of dietary treatments on the α‐diversity indices is presented in Figure 1. Based on Chao's α‐diversity index, no significant differences (*p* > 0.05) were observed in microbiota richness among fish fed CON and AMG diets (Figure 1a). Similarly, Shannon (Figure 1b) and Simpson (Figure 1c) indices showed no significant differences (*p* > 0.05) in bacterial community composition of fish from the different treatments.

**Figure 2:**
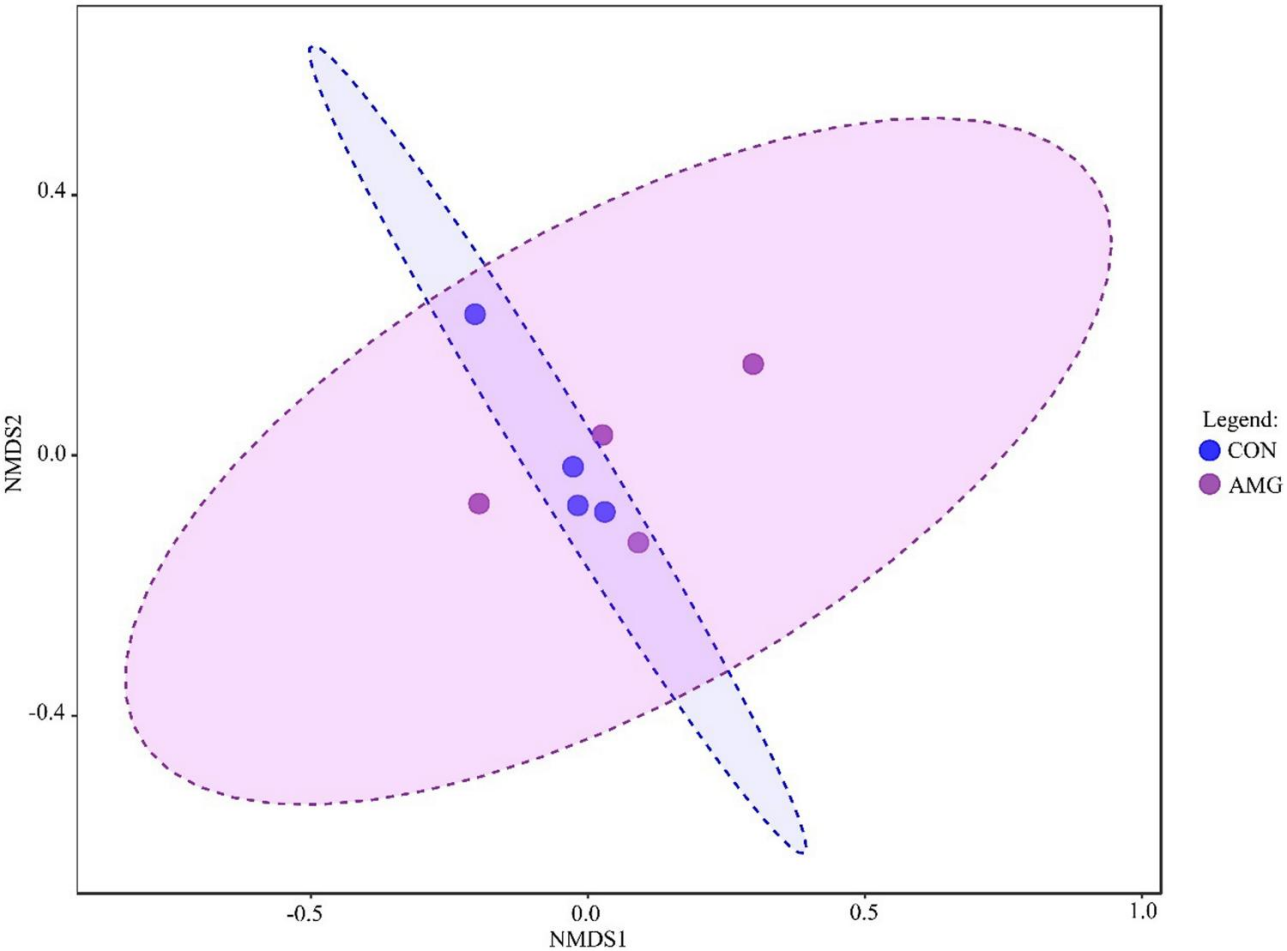
Βeta diversity of juvenile Nile tilapia fed diets either without supplementation (CON) or supplemented with a blend of l‐glutamine and l‐glutamic acid at 20 g kg^−1^ as AminoGut (AMG) over a 60‐day feeding period, evaluated using PERMANOVA (*F*‐value = 0.819; R‐squared = 0.120; *p* = 0.535) and visualized through a Non metric multidimensional scaling (NMDS) plot. NMDS Stress = 0.078. Data for each treatment represent the mean of four replicate aquaria. [Color figure can be viewed at wileyonlinelibrary.com]

The impact of dietary treatments on β‐diversity is shown in Figure 2. The PERMANOVA analysis on the Bray‐Curtis matrix did not reveal distinct clustering (*p* > 0.05) of the gut microbiotas of fish fed CON and AMG diets, which was also confirmed by the NMDS analysis along axes 1 and 2.

**Figure 3:**
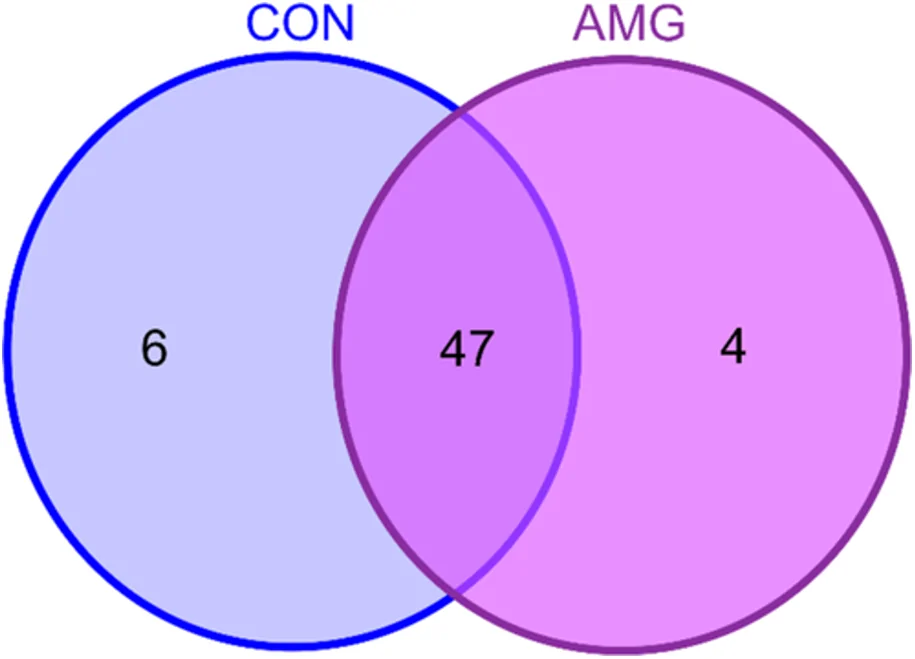
Venn diagram showing the number of exclusive and shared operational taxonomic units (OTUs) of juvenile Nile tilapia fed diets either without supplementation (CON) or supplemented with a blend of l‐glutamine and l‐glutamic acid at 20 g kg^−1^ as AminoGut (AMG) over a 60‐day feeding period. Data for each treatment represent the mean of four replicate aquaria. [Color figure can be viewed at wileyonlinelibrary.com]

The Venn diagram in Figure 3 shows the number of unique and shared OTUs across the dietary treatments. A total of 57 bacterial OTUs were found in the samples. Fish fed the CON diet showed 53 distinct taxa, whereas those fed the AMG diet showed 51. Among these, 47 taxa were common to both groups, while 6 were exclusive to fish fed the CON diet, and 4 were exclusive to fish fed the AMG diet.

**Figure 4:**
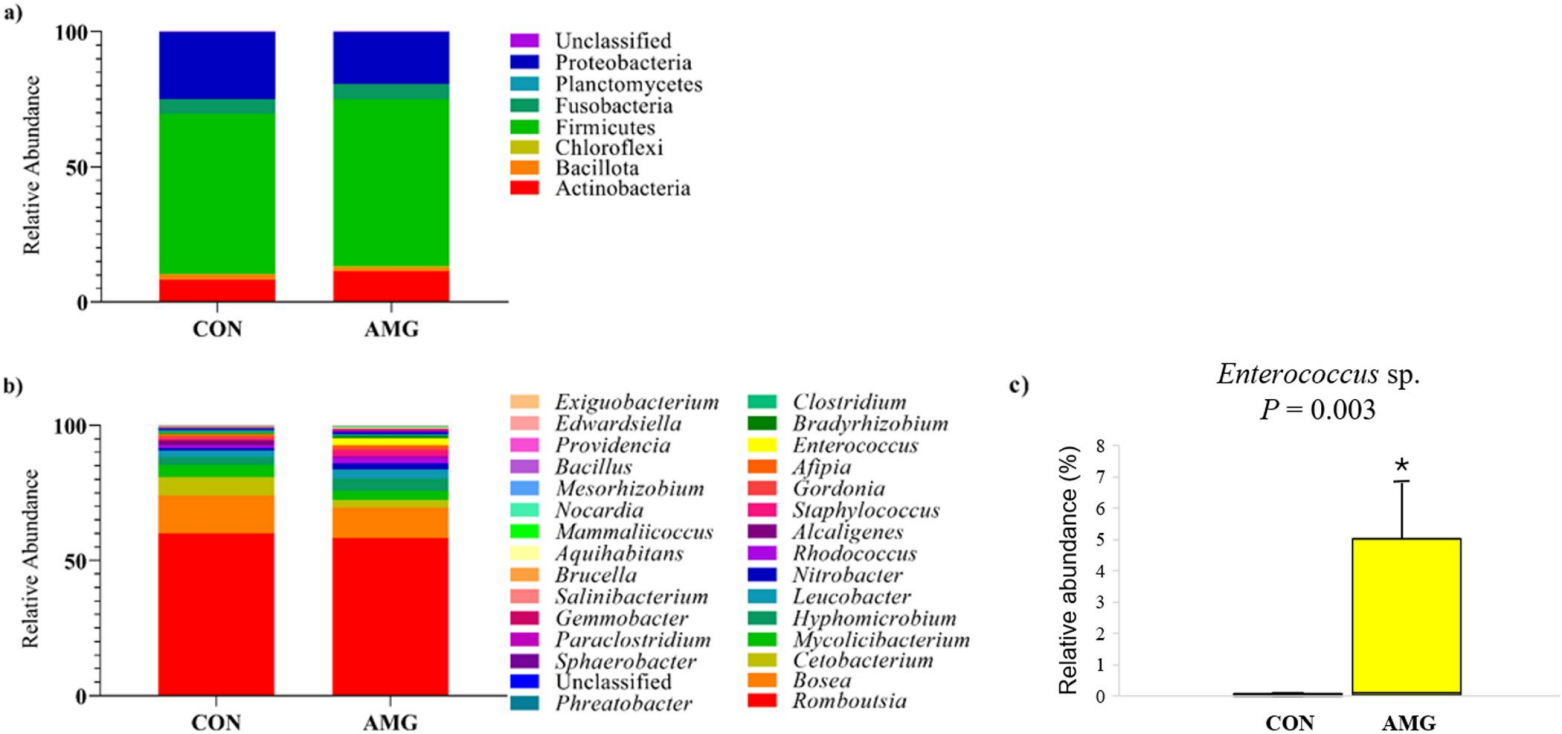
Relative abundance of dominant taxa identified at phylum (a) and genus (b) levels in juvenile Nile tilapia fed diets either without supplementation (CON) or supplemented with a blend of l‐glutamine and l‐glutamic acid at 20 g kg^−1^ as AminoGut (AMG) over a 60‐day feeding period. Data for each treatment represent the mean of four replicate aquaria. Asterisk (*) above bars indicates significant differences by the DESeq2 method (*p* < 0.05). [Color figure can be viewed at wileyonlinelibrary.com]

The relative abundance of bacterial phyla and genera in fish fed CON and AMG diets is shown in Figure 4a,b, respectively. Phyla Firmicutes, Proteobacteria, and Actinobacteria had the highest relative abundance in the digesta samples of fish, irrespective of the diet (Figure 4a). Firmicutes were primarily composed of bacteria by the genera *Romboustia* sp. and *Bosea* sp. Among the Proteobacteria genera, *Cetobacterium* sp. was the most abundant, while in Actinobacteria, *Mycolicibacterium* sp. was the most abundant (Figure 4b). However, the only bacterial genus that differed significantly (*p* = 0.003) in the samples was *Enterococcus sp*. (Firmicutes), which was more abundant in fish fed the AMG diet (Figure 4c).

### 3.5 SCFAs and pH

Table 7 shows the effects of dietary treatments on digesta SCFA concentration and pH. Fish fed the AMG diet displayed higher propionic (*p* = 0.026) and a tendency for higher levels of butyric acid (*p* = 0.096) concentration than those fed the CON diet. However, dietary treatments did not affect (*p* > 0.05) the concentration of acetic acid. Conversely, fish fed the AMG diet presented lower digesta pH (*p* = 0.010) than those fed the CON diet.

**Table 7:** Short‐chain fatty acids and gut digesta pH of juvenile Nile tilapia fed the experimental diets for 60 days^c^.

Parameter	Diet^d^		*p* value
	CON	AMG	
Short‐chain fatty acids (nmol L^−1^)			
Acetic acid	11.27 ± 0.66	12.56 ± 0.31	0.131
Propionic acid	1.37 ± 0.10^b^	1.70 ± 0.04^a^	0.026
Butyric acid	1.17 ± 0.08	1.39 ± 0.07	0.096
pH	7.66 ± 0.04^a^	7.27 ± 0.07^b^	0.010

^a‐b^ Different letters in the same row indicate significant differences by *t*‐test (*p* < 0.05);

^c^ Values are presented as means and standard error of the means of four replicate aquaria (*n* = 4 fish per aquarium);

^d^ Diets were either unsupplemented (CON) or supplemented (AMG) with a blend of l‐glutamine and l‐glutamic acid at 20 g kg^−1^.

### 3.6 Digestive Enzyme Activity

Digestive enzyme activities are shown in Table 8. No significant differences (*p* > 0.05) in amylase, protease, and lipase activities were observed among fish fed the different dietary treatments.

**Table 8:** Digestive enzyme activity of juvenile Nile tilapia fed the experimental diets for 60 days^a^.

Activity (U mg protein^−1^)	Diet^b^		*p* value
	CON	AMG	
Amylase	25.11 ± 0.40	25.45 ± 0.20	0.478
Protease	26.77 ± 0.20	26.15 ± 0.72	0.442
Lipase	3.66 ± 0.11	3.64 ± 0.11	0.918

^a^ Values are presented as means and standard errors of the means of four replicate aquaria (*n* = 4 fish per aquarium);

^b^ Diets were either unsupplemented (CON) or supplemented (AMG) with a blend of l‐glutamine and l‐glutamic acid at 20 g kg^−1^.

### 3.7 Gut Histomorphometry

Table 9 displays the effects of dietary treatments on gut histomorphometry of juvenile Nile tilapia fed the experimental diets. Fish fed the AMG diet revealed higher fold height (*p* = 0.025), a tendency for higher fold width (*p* = 0.075), and a higher fold height: width ratio (*p* = 0.008) than those fed the CON. However, the area of absorption (*p* = 0.517) remained unaffected by dietary treatments.

**Table 9:** Intestinal histomorphometry (µm) of juvenile Nile tilapia fed the experimental diets for 60 days^c^.

Parameter	Diet^d^		*p* value
	CON	AMG	
Fold height	130.92 ± 8.78^b^	157.32 ± 1.30^a^	0.025
Fold width	55.51 ± 1.43	49.52 ± 2.40	0.075
Height: width	2.35 ± 0.12^b^	3.20 ± 0.18^a^	0.008
Area of absorption (µm^2^)	72.99 ± 626.01	7781 ± 314.03	0.517

^a‐b^ Different letters in the same row indicate significant differences by *t*‐test (*p* < 0.05);

^c^ Values are presented as means and standard error of the means of four replicate aquaria (*n* = 3 fish per aquarium);

^d^ Diets were either unsupplemented (CON) or supplemented (AMG) with a blend of l‐glutamine and l‐glutamic acid at 20 g kg^−1^.

### 3.8 Gene Expression

**Figure 5:**
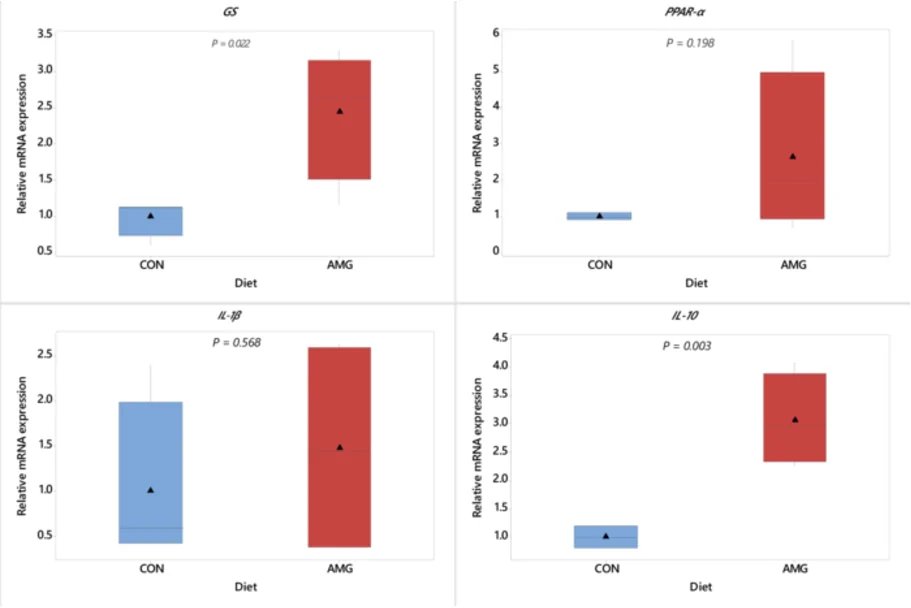
Box plot of the relative *mRNA* expression of liver *glutamine synthetase* (*GS*), *peroxisome proliferator‐activated receptor‐α* (*PPAR‐α*), *interleukin‐1β* (*IL‐1β*), and *interleukin‐10* (*IL‐10*) in juvenile Nile tilapia fed diets either without supplementation (CON) or supplemented with a blend of l‐glutamine and l‐glutamic acid at 20 g kg^−1^ as AminoGut (AMG) over a 60‐day feeding period. Data for each treatment represent the mean of four replicate aquariums. Means were compared by the Kruskal−Wallis test complemented by Dunn's test (*p* < 0.05). [Color figure can be viewed at wileyonlinelibrary.com]

The effects of dietary treatments on gut mRNA levels are presented in Figure 5. Fish fed AMG diet displayed upregulated expression of *GS* (*p* = 0.022) and *IL‐10* (*p* = 0.003) genes than those fed CON diet. However, the *mRNA* levels of *PPAR‐α* (*p* = 0.198) and *IL‐1β* (*p* = 0.568) genes were not affected by dietary treatments.

### 3.9 Oxidative Enzymes and MDA

Table 10 presents the effects of dietary treatments on the activity of oxidative enzymes and MDA concentration in fish liver. Notably, fish fed diet AMG presented higher activity of SOD (*p* = 0.003) and GST (*p* = 0.013) enzymes and lower MDA concentration than those fed diet CON. Conversely, CAT activity (*p* = 0.483) was unaffected by dietary treatments.

**Table 10:** Antioxidant activity and malonaldehyde concentration (µM mg protein^−1^) in liver of juvenile Nile tilapia fed the experimental diets for 60 days^c^.

Parameter	Diet^d^		*p* value
	CON	AMG	
Superoxide dismutase	9.83 ± 0.39^b^	13.22 ± 0.57^a^	0.003
Catalase	1288.24 ± 101.61	1170.92 ± 119.62	0.483
Glutathione‐S‐transferase (µmol min^−1^ g^−1^)	4.34 ± 0.53^b^	9.19 ± 1.27^a^	0.013
Malonaldehyde	10.44 ± 1.64^a^	3.00 ± 1.06^b^	0.009

^a‐b^ Different letters in the same row indicate significant differences by *t*‐test (*p* < 0.05);

^c^ Values are presented as means and standard error of the means of four replicate aquaria (*n* = 3 fish per aquarium);

^d^ Diets were either unsupplemented (CON) or supplemented (AMG) with a blend of l‐glutamine and l‐glutamic acid at 20 g kg^−1^.

## 4 Discussion

This study showed increased biomass in fish fed a diet supplemented with a Gln and Glu blend, aligning with previous results obtained in our laboratory in Nile tilapia fingerlings (Carneiro et al. 2025). The higher survival rate and the numerical increase in body weight gain likely contributed to this key practical parameter response. These improvements can be attributed to the role of these functional AAs in improving intestinal barrier function and strengthening the immune system (Carvalho et al. 2023, 2018; Pereira et al. 2017; Wang et al. 2025), as well as in improving the antioxidant response, as previously reported for juveniles of the species (Carvalho et al. 2023; Wang et al. 2025). These findings are also similar to previous studies conducted in our laboratory, which showed improved growth (Macêdo et al. 2021) and survival (Graciano et al. 2014) in Nile tilapia fingerlings fed the same commercial blend evaluated in this study. Another plausible hypothesis is that Gln and/or Glu modulated the composition of gut microbiota, potentially enhancing digestion and absorption, metabolism, innate immune response, and energy homeostasis of fish (Butt and Volkoff 2019) in the current research.

Regardless of diet, Firmicutes, Proteobacteria, Actinobacteria, and Fusobacteria, respectively, dominated the gut microbiota of fish herein. Previous studies have reported that these bacterial phyla are traditionally components of the core microbiota of Nile tilapia (Deng et al. 2022; Wang et al. 2025), including fingerlings fed the same commercial product used in the current research (Carneiro et al. 2025). However, in fingerlings, Actinobacteria, Firmicutes, Proteobacteria, and Fusobacteria, respectively, dominated gut microbiome. These differences may be attributed to fish size and/or rearing system (Dai et al. 2021; Deng et al. 2022). Considering bacterial genera abundance, fish fed diet with blend of Gln and Glu showed higher abundance of *Enterococcus sp*. (Firmicutes) in the intestinal microbiota. *Enterococcus sp*. are lactic acid bacteria commonly found in the gastrointestinal tract of warmwater fish species, including Nile tilapia (Merrifield et al. 2014). It is a highly diverse genus encompassing both probiotic and pathogenic strains (Krishna et al. 2022). The higher relative abundance of *Enterococcus sp*. resulting from dietary Gln and Glu supplementation is likely related to improvements in the intestinal epithelial barrier, an important factor for the adhesion and multiplication of these bacteria (El Jeni et al. 2020). This assertion is corroborated by the improvement in intestinal SCFA production and histomorphometry in fish fed the diet supplemented with Gln + Glu herein.

This study found that fish fed a Gln and Glu‐containing diet exhibited significantly increased production of acetic acid and propionic acid, with a trend toward higher butyric acid production, which reduced intestinal pH. A previous study in our laboratory reported that SCFAs derived from intestinal bacteria fermentation play a crucial role in intestinal health and function in Nile tilapia (Cruz et al. 2024). Specifically, SCFAs inhibit the growth of potentially pathogenic bacteria by lowering the intestinal pH and providing energy for mucosal cells in fish (Hoseinifar et al. 2017; Tran et al. 2020). Two main mechanisms may explain the enhanced intestinal fold length observed in this study. First, butyric acid serves as an energy source for the development of intestinal cells (Hamer et al. 2008). Second, Gln is deaminated in enterocytes to form glutamate (McCauley et al. 1998), which can be converted to α‐ketoglutarate, entering the citric acid cycle to generate ATP as an energy source for enterocytes (DeBerardinis et al. 2007). Although dietary Gln contributed to intestinal gut development in this study, their supplementation did not influence the activity of digestive enzymes, suggesting that both CON and AMG diets met the dietary requirements of fish. However, whether Gln and Glu promote the relative abundance of *Enterococcus sp*. associated with SCFA production remains poorly documented in the literature and should be investigated in future studies.

Unlike in a previous study conducted in our laboratory in Nile tilapia fingerlings (Carneiro et al. 2025), the expression of *PPAR‐α* gene remained unaffected by dietary treatments herein. In contrast, a previous study reported that Gln supplementation upregulated the expression of *PPAR‐α* gene in the intestine of juvenile grass carp, C*tenopharyngodon idella* (Qu et al. 2019). *PPAR‐α* is regarded as a key regulatory gene involved in fatty acid and AA metabolism in fish, as observed in zebrafish, *Danio rerio* (Li et al. 2020). Notably, *PPAR‐α* activation has been shown to regulate carbohydrate metabolism and modulate energy homeostasis among nutrients, exerting hypolipidemic effects by promoting mitochondrial and peroxisomal fatty acid β‐oxidation in Nile tilapia fed a high‐lipid diet (Ning et al. 2016). Furthermore, dietary Gln was found to downregulate the expression of *fatty acid synthase* (*FAS*) gene in the liver of Atlantic cod, *Gadus morhua*, suggesting a metabolic shift favoring β‐oxidation of fatty acids over triglyceride storage in the liver (Ingebrigtsen et al. 2014). Although in the present study fish fed a Gln and Glu‐containing diet tended to show lower plasmatic triacylglycerol and cholesterol contents and enhanced protein retention efficiency, the expression of *PPAR‐α* remained unaffected. These discrepancies may be related to experimental conditions and fish size used in the present research. However, the plasmatic biochemical parameters and body composition of fish indicate that Gln and/or Glu favored utilization of carbohydrates and lipids as energy sources, thereby reducing AA catabolism for energy purposes while promoting protein synthesis.

In the current study, dietary supplementation of Gln and Glu upregulated the expression of *GS* and *IL‐*10 genes, consistent with previous results found in Nile tilapia fingerlings fed the same commercial AA blend (Carneiro et al. 2025). The increase in the *GS* gene expression indicated enhanced activity of the GS enzyme, which potentially contributed to ammonia detoxification by facilitating its conversion to Gln (Ip 2010; Li et al. 2023). On the other hand, the upregulated expression of anti‐inflammatory cytokine *IL‐10* in the intestine of fish confirmed the protective role of these AAs against intestinal inflammation in Nile tilapia, which is in agreement with results previously reported in Jian carp, *Cyprinus carpio* var. Jian, fed a Gln‐supplemented diet (Jiang et al. 2017). *IL‐10* is an important cytokine produced by immune cells, including monocytes, macrophages, and T cells (Sabat et al. 2010), and develops strong action against inflammatory responses (Fiorentino et al. 1991). Notably, Gln is quantitatively the most important energy source for T cell lymphocytes of mammals (Wasinski et al. 2014) and Nile tilapia (Li et al. 2023). Recent work identified that Gln plays crucial role in immunological responses of leukocyte function in Nile tilapia (Carvalho et al. 2018). Furthermore, an early study demonstrated that *IL‐10* enhanced mIgM+ B lymphocyte‐mediated phagocytosis of *Edwardsiella tarda* in flounder, *Paralichthys olivaceus* (Yang et al. 2019). The role of *IL‐10*, promoting immunoglobulin (IGM) production and activating B cells, has also been demonstrated in Nile tilapia (Wu et al. 2021). However, whether Gln and Glu impact IGM production needs further investigation in Nile tilapia. These may explain the high survival rate in fish fed Gln and Glu‐containing diets in the present study. These provide insights into the roles of Gln and Glu in enhancing innate immunity, implicating the evolutionary conservation of this proinflammatory cytokine in Nile tilapia.

The current study evidenced that fish fed a Gln and Glu blend showed higher activity of GST and SOD and reduced hepatic MDA concentration. GST activity serves as a valuable biomarker to assess the antioxidant status of fish, as this enzyme facilitates the conjugation of GSH with potentially toxic compounds and reactive oxygen species (Ketterer 1998). The increase in the activity of GST in the liver may be associated with higher availability of GSH, composed of glutamate derived from Gln or Glu, cysteine, and glycine (Harington and Mead 1935). An earlier study reported that Glu increased GST activity and attenuated oxidative damage to tight junction and defensin proteins induced by lipopolysaccharide in Jian carp (Jiang et al. 2017). Meanwhile, it has been reported that Gln enhanced SOD activity in saponin‐induced enteritis in turbot (Gu et al. 2021). Furthermore, Gln supplementation has been demonstrated to upregulate SOD gene expression and consequently reduce MDA concentration in enteropathy induced by high dietary soybean in turbot (Liu et al. 2015). These results demonstrated that Gln and Glu may enhance the antioxidant response by increasing the activity of GST and SOD enzymes, thereby improving oxidative stress resilience in Nile tilapia.

In the present study, Gln and Glu supplementation increased the plasmatic ALP activity. This finding aligns previous results obtained in chicken (Wu et al. 2021). Higher ALP plays a crucial role in osmoregulation, cellular energy metabolism, and in transportation of AAs, fatty acids, calcium, and phosphorus in the intestine (Sanderson and He 1994). Plasma ALP originates from the liver and generally increases due to bile duct impairment or obstruction (Yong 1967). Recent studies have evidenced more complex functions of ALPs in human, rat, and zebrafish health (Rader 2017). ALP deficiency in the intestine has been directly linked to intestinal inflammation in humans and rats (Tuin et al. 2009) and zebrafish (Bates et al. 2007). It has been hypothesized that the protective role of ALP may include its role in gut microbiome shaping and homeostasis (Malo et al. 2010). These findings highlight the protective role of Gln and Glu against intestinal inflammation, as evidenced in the current study by increased *IL‐10* expression and microbiome‐related mechanisms. These findings underscore the potential of Gln and Glu as functional AAs in Nile tilapia juveniles, highlighting their role in enhancing growth, gut health, and function, and overall well‐being, thereby offering promising benefits for sustainable and efficient production practices.

## 5 Conclusions

Dietary Gln and Glu supplementation at 20 g kg^−1^ promoted growth performance and survival of Nile tilapia juveniles by modulating gut microbiota and sustaining the intestinal mucosa development and function. These effects supported improved nutrient metabolism, activation of innate immunity, and increased antioxidant capacity of fish, highlighting the potential of Gln and Glu as functional AAs in Nile tilapia aquaculture.

## Ethics Statement

The authors confirm that the ethical policies of the journal, as stated on its author guidelines page, have been followed. Appropriate approval from the ethics review committee was obtained. This research was approved by the Ethics Committee of the State University of Ponta Grossa (CEUA/UEPG) under protocol number 23.000022613‐5, in accordance with the guidelines of Normative Resolution CONCEA No. 52, dated May 19, 2021 (Brazil).

## Conflicts of Interest

The authors declare no conflicts of interest.

## Supporting information

Supporting File

## Data Availability

The data that support the findings of this study are available from the corresponding author upon reasonable request.
