# Supplementary material for: L‐Glutamine Plus L‐Glutamic Acid Enhances Antioxidant Status and Ammonia Toxicity Resilience, Upregulates Interleukin IL‐10 Gene, and Improves Gut Microbiota and Survival in Juvenile Nile Tilapia
Source: J Anim Physiol Anim Nutr (Berl). 2026 Jun 28;110(5):715–30. doi: 10.1111/jpn.70086 (PMC13576543; doi:10.1111/jpn.70086)
Supplement: Supplementary file 1 [file JPN-110-715-s001.docx]

**Supplementary material and methods**

**Figure S1.** Rarefaction curves of Chao and estimators of different replicate samples in juvenile Nile tilapia fed diets either without supplementation (CON) or supplemented with mixture of L-glutamine and L-glutamic acid at 20 g kg^−1^ (AMG), over 60 days feeding trial. Data for each treatment represents the mean of four replicate aquariums.
